# Walk for Mental Well‐Being as a *Third Place*: A Qualitative Study of the Bathurst Men's Walk and Talk

**DOI:** 10.1111/ajr.70066

**Published:** 2025-06-23

**Authors:** Peter Simmons, Uchechukwu Levi Osuagwu, Hazel Dalton, Julaine Allan, John Eme, Tracy Macfarlane, Haider Mannan

**Affiliations:** ^1^ Rural Health Research Institute Charles Sturt University AU Orange New South Wales Australia; ^2^ Gulbali Research Institute Charles Sturt University AU Albury New South Wales Australia; ^3^ Bathurst Rural Clinical School Western Sydney University AU Bathurst New South Wales Australia; ^4^ Translational Health Research Institute (THRI) Western Sydney University Campbelltown AU Campbelltown New South Wales Australia

**Keywords:** community‐based, mens mental well‐being, rituals, rural, third place, walk group

## Abstract

**Objective:**

To explore processes that engage rural men in a weekly, enduring, community‐volunteer‐organised walk for mental well‐being, and compare with characteristics of *Third Places* (relaxing social places that are neither home nor work).

**Setting:**

Bathurst, regional city population 44 939 (2024), New South Wales, Australia.

**Participants:**

Bathurst Men's Walk and Talk (BMWT) walkers tend to be older and in a married/defacto relationship. More than one‐third score low on mental well‐being indices.

**Design:**

In‐depth semi‐structured interviews (*n* = 20) with walkers (13) and leaders (7) of the BMWT were thematically analysed, inductively and deductively.

**Main Outcome Measures:**

Interviewee descriptions of experiences of BMWT and its impacts reported against Oldenburg's (1989/1999) eight characteristics of *Third place*, and use of rituals.

**Results:**

BMWT is more routinised but has essential characteristics of an ideal *Third place*. BMWT accentuates the essence of *Third place* including welcome, inclusion, conversation and belonging to the group. BMWT balances culture and structures that meet diverse needs for connection and enjoyment in the manner of a *Third place*, while communicating safety and reassurance required for men who seek or need support for mental well‐being. Interviewees reported mood and well‐being benefits from BMWT physical activity, social interaction and belonging.

**Conclusion:**

Thoughtful planning can increase health‐giving social interaction and feelings of belonging consistent with *Third place* experiences. In rural areas where men often miss out on mental well‐being support, *Third place* provides a framework to guide individual group and community planning.

**Summary**

- Practitioner points
  ○ Social and group factors drive continued participation in well‐being groups.
  ○ *Third place* features such as levelling, welcome, conversation and playfulness can heighten enjoyment and participation.
  ○ Rituals can enhance opportunities for interaction and feelings of safety and belonging to the group.
- What is already known on this subject
  ○ Rural men experience worse mental health outcomes than men in the general population, are less likely to use mental health services or seek support, and National priorities for mental health promotion include gender‐specific interventions.
  ○ Community‐based well‐being programmes can provide social interaction and feelings of belonging that complement health services and mitigate harm from rural resource shortages.
  ○ Life circumstances and health‐related events often prompt people to join community groups, but the decision to continue is based more on social and group cohesion factors.
  ○ *Third place* provides a framework that enables analysis of social and group factors in a community setting.
- What this paper adds
  ○ Bathurst Men's Walk and Talk is an inclusive volunteer‐run programme balancing a culture that provides connection and enjoyment in the manner of *Third place*, while communicating trustworthiness required for men who seek or need support for mental well‐being.
  ○ Rituals such as free coffee, pre‐walk briefing and half‐way photo‐stop repeatedly bring the group together and facilitate conversation, increasing social interaction and feelings of belonging.
  ○ In rural areas where men in need often miss out on mental well‐being support, *Third place* provides a framework to guide planning for individual groups and communities.

## 1 Introduction

Australia's National Men's Health Strategy 2020–2030 highlights socially isolated and rural men as priority populations due to higher rates of mental ill‐health, suicide and barriers to accessing services [1]. The strategy calls for innovative approaches, including male‐focused outreach, community services and health literacy interventions [1]. Bathurst Men's Walk and Talk (BMWT) is a rural community‐run programme taking up the challenge of supporting men's mental well‐being. Participation in BMWT has steadily increased over 5 years, this paper examines its approach to engaging rural men against characteristics of *third places*. The *third places* framework [2, 3] is used internationally in civic planning to address loneliness and healthy ageing [4, 5].

Rural men experience diverse struggles across intersecting factors such as race, class and cultural background, and we need to avoid incorrect and harmful stereotyping [6]. However, rural men are often less likely to access well‐being groups and mental health services, and gender‐specific approaches to mental health help‐seeking are recommended [7]. Social interaction has been found to be especially beneficial following significant events such as retirement or partner illness [8], and for vulnerable groups, including rural men [9]. Older people can miss out on social interactions because of retirement, or mobility, sight or hearing decline [10], necessitating new opportunities for connection.

Walking is popular with older people maintaining physical and mental health, and group walks expand social networks [11, 12]. However, men are less likely to participate in walking groups [13] or community‐based exercise programmes in rural areas [14]. While life and health‐related events often prompt people to join such groups [12, 13] continued participation depends on social factors like friendliness, safety, enjoyment [12], belonging, trust [15] and group cohesion [16]. These factors align with characteristics of successful third place*s*, where welcome and conviviality matter more than the physical space [3].

Informal community‐based approaches are crucial for rural men's mental health [9] and *third places* outside home and work have been suggested as potentially important settings for providing social interactions that can mitigate loneliness [5, 17] and increase belonging [18]. Traditionally physical, but increasingly also virtual [5], *third places* are accessible, unpretentious and often single‐gender [3].

*Third places*, and how to find them, have been a focus in recent Australian media [19, 20]. *Third place*, refers to publicly accessible places for relaxation where people feel comfortable to be themselves and share personal information, but they are separate from home (first place) and school/work (second place) [3]. *Third places* are ‘the people's own remedy for stress, loneliness, and alienation’ [3], where leisurely conversation and informal social interaction are the main activity [21], and good‐natured banter, among (unrelated) regulars is normal [22]. An Australian study emphasised the provision of readily accessible *third places* to promote social health and well‐being among older people [23]. A Swedish study found that community, safety, affordability, nature and independence in *third places* help combat loneliness and improve well‐being [5]. Cafes [22], bars, churches, parks [24], libraries [25], community gardens [21] and gyms [26] have all been described as third places. Dolley [21] highlights value to the community from social diversity within *third places*, and distinguishes the public inclusivity of *third places* from the closed exclusivity of clubs and societies with membership criteria. Others emphasise that *third place* arises from an accumulation of interactions and feelings associated with those interactions, more than a defined physical place [27]. The value of *third places* in rural areas, particularly those experiencing economic decline and institutional loss, cannot be overstated [28, 29].

This paper focuses on processes used by an enduring community‐volunteer‐run walk, the *Bathurst Men's Walk and Talk* (BMWT), to engage rural men and support mental well‐being. Given the place‐based nature of the programme, BMWT has been analysed against the theoretical framework and characteristics of *third place*.

## 2 Methods

This paper reports the qualitative findings of an evaluation study of BMWT. The in‐depth interviews reported here explored participants' experiences, a separate survey profiled walkers' mental well‐being and attitudes [30]. After initially participating in one walk as observers, two researchers regularly joined BMWT walks and were included in the group's associated activities and communication. The interview study thus became more ethnographic [31] and emic [32] than a normal interview study as these researchers spent extended amounts of time with the researched, developed relationships and trust, and shared experiences with the walkers [33]. This closeness to the data and lived experience can enhance the richness of data and interpretation [33]. This paper focuses on data gathered from the qualitative interviews.

### 2.1 Programme Description—Bathurst Men's Walk and Talk (BMWT)

BMWT is a weekly riverside walk promoting men's mental well‐being through exercise and social interaction. It has operated since 2019, pausing only during strict COVID‐19 restrictions. It is run by volunteers and auspiced by Lifeline Central West who provide public liability insurance and mental health awareness training to group leaders (known as Yellow‐Caps). Yellow‐Caps are not health professionals; when required, they provide information about where to get more mental or other health information and support. Three of the six founding Yellow‐Cap leaders still walk most weeks. New Yellow‐Caps are recruited as vacancies arise or group numbers grow; they are men who have shown commitment to BMWT through regular attendance and demonstrate interpersonal sensitivity and skills.

Every Thursday at 17:30, in all weathers, men gather for a free hot drink and informal chat before walking at 18:00 for an hour, just under five kilometres. Participants mostly walk in pairs, underscoring BMWT's motto, *Never Walk Alone*. Although there is a small decline in participation in colder months, each year weekly attendance has steadily grown from 4–6 in 2019 to 20–30 in early 2025. A total of about 140 men have participated at least once.

Bathurst is a small regional city (population 44 939) [34] with a higher proportion (10.9%) [35] of people experiencing mental health conditions (including depression and anxiety) than New South Wales (8%) [36] or Australia (8.8%) [37]. BMWT has developed considerable local goodwill, as reflected in positive publicity and business sponsors paying for T‐shirts and coffee. The group communicates regularly among walkers using WhatsApp, and outwardly through social media, in particular the group's Facebook page. Recruitment tends to be from Facebook awareness raising, local media and ‘informal networking’ [31], with occasional referrals from health professionals.

A survey study (*n* = 40) of BMWT found that BMWT walkers are mostly older (75% aged above 50) and a large proportion (43.2%) had tertiary education qualifications, compared with New South Wales (27.8%) [37]. Many face or may be considered at risk of mental ill health according to scores on validated mental well‐being indices. Forty‐two per cent scored below the social isolation threshold on the Duke Social Interaction Subscale [38] and 26% scored below the cut‐off for poor mental well‐being on the World Health Organization Five‐Item Well‐Being Index [39]. A majority (68.4%) of BMWT walkers had experienced one significant, negative life event in the past 12 months and nearly a quarter (23.6%) had experienced three or more events. Compared to the wider population, they were three times more likely to have experienced the death of a close relative or family member, twice as likely to have experienced the death of a close friend, six times more likely to have had a serious personal injury or illness, and six times more likely to have retired from the workforce. A large majority of walkers said that BMWT is important or very important to their mental well‐being (80%), physical well‐being (82.5%) and social well‐being (75%) [1].

### 2.2 In‐Depth Interviews

#### 2.2.1 Recruitment Process

Ethics approval was obtained from Western Sydney University HREC number H15627 ensuring that all research procedures complied with Helsinki's Declaration for research on human participants. All participants provided written informed consent prior to participating in the study. Most interviewees were recruited via survey invitations, with contacts provided separately to their anonymous survey data. Researchers directly emailed auspicing managers and three younger walkers to increase the age diversity; all accepted. Interviewees received details of the study aims and interviewer, a $60 voucher, and assurances of confidential participation. They were repeatedly reminded that participation was voluntary and that they could withdraw for any or no reason.

#### 2.2.2 Interview Process

Sixty‐minute semi‐structured interviews were conducted (Feburary–April 2024) by five experienced male and female researchers at convenient locations, mostly workplaces and homes. A question guide (Appendix S1) included planned prompts. Walkers discussed participation motivations, likes, dislikes and key walk features. Leaders discussed BMWT features and rituals and meeting participant needs. Three interviewers (PS, LO, HD) conducted leader interviews; four (PS,LO, EJ, TM) interviewed walkers.

#### 2.2.3 Data Analysis

Interviews were audio recorded, professionally transcribed and interviewees were invited to review transcripts. A multistage thematic analysis helped to deepen understanding of BMWT and its impacts [40]. Interviewers documented key responses in shared matrices using notes including direct quotes. Two (PS, EJ) independently summarised data, noting recurring themes (e.g., ‘liking shirt as uniform’) and broader concepts (e.g., ‘belonging’) [41]. Four researchers (PS, EJ, LO, TM) finalised major and minor themes through consensus. Analysis was initially inductive but later structured deductively around themes introduced to the interviews as standardised questions on topics of prior interest [33].

Researchers examined transcripts for examples of ritual and *third place* characteristics. Rituals are repetitive, culturally influenced actions that structure expectations in social contexts [42, 43]. Researchers identified commonly described events. *Third place* characteristics [3] were also analysed, summarised in Table 1.

**TABLE 1:** Characteristics of *Third Place* [3].

Characteristic	*Third Place* ‘ideal’ [3]
Neutral place	Where people ‘come and go as they please’ [44], feel welcome without host/guest obligations [26].
Leveller	An inclusive place that does not require particular criteria for membership. Outside status and problems left at door.
Conversation is main activity	Encouraging of and amenable to sociable conversation.
Accessible and accommodating	Convenient to access, handy to home or work.
Regulars	With people who attend frequently, help welcome newcomers, and create a sense of familiarity that is part of the identity of the place [25].
Low profile	Where people commonly meet informally.
Playful mood	Encourages spontaneous wit and humour and break from life stresses [45].
Home away from home	Familiar places where people feel comfortable sharing personal information and free to be themselves.

The analysis also draws on Dolley [21], Vaux and Langlais [45] and others who affirm Oldenburg's [3, 44] criteria remain relevant but require updates for a digital world, and places less frequently open than churches or coffee shops [26].

## 3 Results

### 3.1 Sample

Seven group leaders (including five Yellow‐Caps) and 13 regular walkers were interviewed. Table 2 shows a spread of ages, but most interviewees (80%) were aged over 50. Most were in married/defacto relationships (90%) and employed (80%). Most (85%), including all leaders, had been involved with BMWT for more than 6 months.

**TABLE 2:** Interview sample demographics.

Interviewees (*n* = 20)						
Gender	BMWT role	Relationship status (*n*)	Employment	Age range		Time with BMWT
Male (19) Female (1)	Walkers (13) Walk leaders (7) Yellow‐Caps (5) Auspice body (2)	Married/defacto(18) Separated (1) Widowed (1)	Employed (16) Retired (4)	18–29 30–39 40–49 50–59 60 plus	1 2 1 9 7	1–6 months 3 > 6 months 17

All of the leaders had lived in Bathurst for more than 20 years, and had extensive community networks through employment, schools and/or sport.

### 3.2 In What Ways Does BMWT Align With Characteristics of a Successful *Third Place*?

This section reports comparisons between BMWT and Oldenburg's [3] ideal of a successful *third place*. Table 3 summarises the findings, showing that BMWT aligns with all of the *third place* criteria. Where BMWT deviates from *third place* ideal it tends to enhance qualities that are the essence of *third place*, such as welcoming and opportunities for interaction (W = Walker:L = Leader).

**TABLE 3:** Comparison of BMWT and ideal *third place*.

Characteristic	*Third Place* ‘ideal’ [3]	Features of BMWT comparision
Neutral place	Homely and comfortable. Feel welcome. No one obliged to play host or guest.	Neutral, public, comfortable, well‐maintained facility and amenities. Free of charge. Free coffee is welcoming. Some hosting by Yellow‐Cap leaders to welcome and orient newcomers, and guide structured BMWT processes. Provide health information. Not run by health professionals.
Leveller	No membership or criteria for inclusion. Contacts expanded beyond normal social circles. Rank, status and problems left at door. Mood is upbeat and cheerful.	No membership or criteria for inclusion. Contacts expanded beyond normal social circles. Rank and status left at door. All men welcome. ‘Never Walk Alone’ on flat, easy path suits most abilities. Two circuits more inclusive than one long circuit. Walkers report diverse backgrounds are enjoyable and broadening. Free T‐shirt uniform is equalising. Welcome to share problems for mental well‐being, but atmosphere generally positive and upbeat. For many BMWT is an escape from routine or problems.
Conversation is main activity	Conversation is central, valued and engaging. Activities facilitate, encourage conversation (e.g., cards, craft).	Conversation is central, valued and engaging. ‘Conversation is most enjoyable part of BMWT’. Walking side‐by‐side in pairs facilitates conversation. Coffee an icebreaker. Coffee, mid‐walk stop, switching partners and post‐walk regrouping facilitate multiple conversations.
Accessible and accommodating	Open long hours, convenient location, unstructured activity, suits unplanned ‘drop in’.	Available just once per week, central location, easily accessible by car. Minimal barriers to joining in. Drop in encouraged, leaders say ‘just turn up’. BMWT structures (routine, rituals) enhance feelings of welcome, safety, predictability.
Regulars	Attendees who feel at home, set tone and welcome newcomers. Regulars' personalities provide familiarity, atmosphere and identity. Accepting culture, delights in accepting unlikely members. Frequency of attendance is criterion for becoming a regular. Seem less diverse from outside than they feel.	Regulars welcome newcomers, provide familiar upbeat atmosphere, and ensure no one walks alone. Frequency of attendance is criterion for becoming a regular. Perceived as more diverse from within than from outside. Less ethnically and culturally diverse than wider community.
Low profile	Plain but homely venue built for other purposes but available for gathering and hanging out. Unpretentious décor and manner of dress.	Covered outdoor picnic area sometimes frequented by homeless people, tourists and strollers. Well maintained, utilitarian barbecue, seating and toilet amenities. Casual dress expected.
Playful mood	Persistent mood is playful, group often laughing and boisterous. Wit and humour, ‘joy and acceptance reign over anxiety and alienation’.	BMWT default group mood is upbeat, positive, and humorous. Individuals choose the tone and content of their conversation interactions. At BMWT dark mood, anxiety or negative life events are discussed appropriately and as required, not in the a group but one‐to‐one.
Home away from home	Like home, grounding in the manner of routine—same place at about the same time each week. Provides a range of psychological supports. Most often places of same gender. A public place for a narrow range of activities. More homely (insider) than visiting someone else's home (outsider). Regenerate the spirit. Freedom to be oneself. Friendly, supportive and warm (more so than some homes). Offers companionship for people from one‐person households.	Same place at about the same time each week. A healthy habit that is part of the routine of the week. Provides a range of psychological supports. Same gender. A public place for walking and talking. Regenerates—mood lifting, energising. Break from routine or pressures of home and work. Friendly, supportive and warm (more so than some homes). Companionship, connection, and conversation. Sense of belonging.

#### 3.2.1 Neutral Place

The BMWT venue is a neutral, welcoming space where people can come and go freely. The well‐maintained park and paths are public and free. BMWT requires relatively little oversight, but BMWT prioritises feelings of welcome and inclusion. Free coffee is welcoming, and Yellow‐Caps play host‐like roles ensuring newcomers welcomed and oriented, no one walks alone and the walk operates smoothly. Although not mental health professionals, most have completed mental health awareness training. Many interviewees mentioned feelings of welcome and inclusion.

They would turn around and see me there and then somehow or other they would drop back and the next thing we're three and they're talking across me but asking me at the same time and including me. I found that I felt more included, I suppose, in the fact that people wanted to talk to me and I was able to talk back and get a bit of interest, I suppose, from them. W3

Most interactions occur without host/guest obligations but Most interactions are informal, but some structure helps newcomers settle in, ensures responsibilities are met, and fosters confidence and safety.

#### 3.2.2 Leveller

Many features of BMWT pertain to social and physical inclusion. Leaders consider it important to minimise barriers to walking, so there is no requirement to register, just a name and phone number kept, and weekly attendance count for coffee payments.

… it's not like a club or membership, it's just turn up. L6

BMWT shirts given free to walkers were described as levellers. Although optional, most wear them. Some said they always preferred to wear uniforms at school and work; they create equality.

If they're wearing the same shirt, you should be able to talk to them on the same level. L5

Flat concrete pathways, seating and toilets cater for diverse physical needs and abilities. The walk's two‐circuit structure allows for late arrivals and early exits, suiting those less fit.

That's the beauty of that walk; it's probably 90 per cent flat … We've had guys that when they first start, they can only do one lap and within a few weeks they're able to do the two. L5

BMWT walkers are encouraged to feel safe to talk about problems, and some do. For others, BMWT camaraderie provides a welcome break from routine stresses, or an escape from grief, and they leave problems at the door.

It's just an avenue for men to get out for an hour and a bit… and just don't have to worry about anything, you know? W8

Walkers get to know other BMWT walkers and their different stories and backgrounds. Many commented on BMWT's openness to all men, regardless of background, placing high value on connecting with diverse people they would not otherwise meet.

Whoever you're talking to, they've had a completely different life experience to you … And it really broadens your understanding and appreciation of the world. W1

Some interviewees, however, recognise that BMWT appears monocultural from the outside:

The walk is very white. Perhaps we could do more to encourage people who are not so like us. W12

A large majority of the BMWT walkers are older white males, and women are not the only sub‐group missing, but it feels to most walkers that BMWT has created a levelling place where the importance of status outside BMWT is diminished.

#### 3.2.3 Conversation Is the Main Activity

BMWT encourages ‘talk’ as the main activity, with rituals facilitating multiple conversations each week. When asked what they like or dislike, most walkers referred to conversations, and about a quarter valued meeting diverse people.

Local businesses sponsor a coffee van offering free hot drinks. Importantly, it arrives 30 min before the walk, encouraging early arrivals and extending ‘talk’ time. Some interviewees noted that free coffee helps newcomers feel welcome, while others said it meets physical needs for stimulation.

It's good. Like an icebreaker, I guess. We stand around now and you're waiting for your coffee and you'll just talk to whoever's beside you. L5

Another ritual involves regrouping on a pedestrian bridge after the first lap, allowing slower walkers to catch‐up and reinforcing the ‘group’. A group photo is taken for sponsors, Yellow‐Caps check if anyone needs help, and men can leave early or arrive late. Although not required, most switch walk/conversation partner, increasing diversity of the experience.

Because walkers regroup at half‐way, complete the second lap closer together and regroup again. On completion, many converse briefly, but seldom longer than 15 min.

Interviewees said BMWT conversations benefit mental well‐being. Some felt reassured they now had a network of men they could rely on if they ever needed help in the future, others that they were not alone in experiencing struggles. One walker described longstanding mental health challenges and difficulty interacting and making friends. He said the *Never Walk Alone* policy helped him to become a better conversationalist.

At the start – probably the first couple of months – they'd have to start the conversation. Then you would learn how to start that conversation yourself with new people … later on when someone new started … I'd go up to them and start the chat which I would never have done before. I find I can still do that now. I can talk to strangers better than I used to be able to. W3

From start to finish, BMWT conversation seldom ceases.

#### 3.2.4 Accessible and Accommodating

BMWT is a structured activity. It is not in a residential or commercial neighbourhood, but the meeting place is well signposted, just 1 km from the town, with ample parking and good access for pedestrians, bikes and cars. The entire walk is off‐road. One leader commented that an easily located venue is crucial for well‐being programmes.

Attending one of these things or going to the GP or whatever. What's so hard, is to get people to take that first step. So, if you have the walk centred around a local landmark, like, it just makes sense, I guess. L2

BMWT occurs every week at the same time and place, encouraging people to ‘just turn up and walk.’ This consistency enhances accessibility.

I like the consistency because you always know what you're going to go to. So if you're 10 min away and you think, ‘Am I going to go to it? Am I not?’ You don't have to think about where are they meeting? How far are they going tonight? Where is it to? It's always the same. It's always consistent. You're always going to know. W11

BMWT is only available once weekly, and mainly suits vehicle owners. But in many important ways it is accommodating.

#### 3.2.5 Regulars

Regular walkers, their personalities, and interactions are the essence of BMWT. They welcome newcomers, providing predictability and gentle formality that builds trust.

We're very mindful that as a group, especially the Yellow‐Caps, if we see someone new, we try and engage with them early. Like, ‘G'day, I'm XXXX, who are you? How did you find us? Have you ordered your coffee yet?’ … we want to be inclusive, we want to be that group where people feel welcomed and want to come into. L6

Three of five 2019 founders still walk most weeks. The main criterion for becoming a ‘regular’ is to attend frequently and BMWT has a growing core of regulars. Approximately 12–15 reliably attend weekly. Absence of these men is noticed and often jokingly commented on. A further 15 or more attend less often.

The coffee van owner is often the first contact; he has an excellent memory for individuals' drink preferences. Three minutes before the walk, a Yellow‐Cap welcomes new walkers, explains the walk, thanks sponsors, often raises awareness of a men's health issue, reinforces respect for others on the path and confidentiality of discussions. The brief pre‐walk chat sets a friendly, respectful tone.

One Yellow‐Cap is a keen timekeeper. Most weeks the group joke about his strictness, but walkers are grateful for consistent start and finish times. Other ‘in‐jokes’ occur at each gathering point. Light‐hearted banter makes the enforcement of routine enjoyable.

Interviewees believe the stable core of regulars will sustain BMWT. One man is the central organiser and driver of BMWT, and replacing him, should he ever leave, is considered the main sustainability challenge.

I think going forward there will always be enough to keep it going. And there's not a huge amount. It's not as though every Saturday morning you have to set up all the goalposts for three hours. L3

BMWT has developed a comforting familiarity that is sustained by interactions and rituals among regulars who welcome others to join them.

#### 3.2.6 Low Profile

BMWT starts and finishes in a low profile outdoor covered eating area with seating for approximately 30 people and ready access to public toilets. Seating is utilitarian but makes waiting more comfortable for those who might otherwise remain in their motor vehicle, and adds to the talk and interaction. Proximity to public toilets is valued by some older walkers. The facilities are also used by the homeless and travellers. Occasionally there is a reminder of remoteness that suggests the venue is more acceptable for male‐only gatherings. There is no need to dress up. The area of the walk is an inauspicious but attractive public greenspace where people gather informally for exercise, picnics and enjoyment.

#### 3.2.7 Playful Mood

Numerous interviewees described good‐natured banter among walkers, and its positive impact on their mood and well‐being. Around half of the interviewees also said that BMWT led them to increase exercise outside BMWT, especially walking. While walkers take health and sponsor matters seriously, the default mood is playful and humorous.

It's just – everybody's happy … we take the Mickey out of each other, but it's all … in a good humour. Nobody takes offence to it … I've been there since the start and there hasn't ever been an argument. There has never been something where somebody has really got stroppy or took a – taken offence to it or anything like that. So it's – it's just a fun group, you know? W8

Interviewees also noted that conduct in interactions is appropriate as required, especially in more emotional one‐on‐one interactions.

… it's a space where blokes can catch up, have a chat, whether that be about the footy, the cricket, the cars, anything. Or if they want a little bit more of a personal chat, the availability is there. L3

Interviewees were inclined to say they value gender‐specific groups, as some will only ‘open‐up’ with their own gender, and the ‘quality’ of banter is different with women present.

#### 3.2.8 Home Away From Home

BMWT is grounding like home in the manner of familiar routine (‘a healthy Thursday walk’; ‘like weekly football training’), where regulars feel at ease. It helps with short‐term companionship needs such as during a partner's custody week, and long‐term reassurance of a dependable group in tough times. Interviewees appreciated that BMWT has continuously provided a ‘safe space’ where men feel comfortable sharing personal information related to mental well‐being.

I was walking with a bloke … he was telling me all about basically his marriage breakdown, and he was losing the kids and going through that process. And you could tell he was on the verge of breaking down every time he was talking. W12

BMWT's mental health focus was said to deter attendance by some men who could benefit. Several interviewees said they would like the community to better understand that BMWT walkers lead regular lives and face common well‐being challenges.

We've evolved the way we approach it and we have it more as just a Men's Walk n Talk and we don't push the mental health side of it so hard as we used to. L1

Most BMWT leaders would like to see more walkers each week, but they appreciate that in a smaller group it is easier to call everyone by name and achieve familiarity and belonging.

I see guys that have left the group and I'm concerned about why. We're not the fit for every man … but I wonder if there's things that we've let go that we could have done better. L6

Leaders accept that they won't meet the needs of all, but regularly reflect on ways to better accommodate and include.

## 4 Discussion

BMWT shares key characteristics of *third place*, balancing culture and structure to meet diverse needs for connection and enjoyment, while communicating safety and reassurance required for men who seek or need support for mental well‐being. For many, BMWT is a relaxing *third place* away from home and work where one can escape life's pressures [46], and enjoy light‐hearted interactions with familiar men who are not relations [22]. BMWT is socially inclusive, requiring no membership, and frequency of attendance is the criterion for becoming a regular [3]. Its public, government‐maintained path and parkland facilities [5] provide a neutral, accessible, affordable, low‐profile venue [3] that is inclusive of most physical abilities. BMWT's unique rituals, regulars and leadership capture *third place* *'s* welcoming, inclusive and playful home‐from‐home essence [3]. Interviewees reported mood and well‐being benefits from BMWT's physical activity, social interaction and belonging [5, 17, 18] and increased exercise at other times in the week, especially walking.

BMWT prioritises mental well‐being support, providing a safe space for men to share concerns. However, leaders perceive stigma from BMWT's explicit mental health association, deterring some who might benefit. BMWT continues to explicitly communicate its central mission concerning mental well‐being support, but since inception in 2019 BMWT has broadened communication beyond ‘mental’ well‐being to promote walking and talking in green space as part of a group that is open to all. Communications emphasise characteristics typical of *third place* *—*enjoyment, welcome, accessibility, inclusion, conversation and belonging [3].

BMWT is more structured than Oldenburg's original (1989) unplanned, freewheeling and spontaneous *third place* ideal. However, the main purposes of BMWT structures and rituals—facilitating social interaction and reinforcing the group—are consistent with the *third place* ideal and well‐being benefits [5]. Rituals tend to be repetitive, culturally influenced actions that provide structure, guiding expectations of what occurs and how to behave in social situations [42, 43]. For some men who are shy or lack social skills, BMWT rituals that repeatedly bring men together and insist that no one walks alone enhance their opportunity to experience the benefits of interaction and belonging.

BMWT prioritises conversation and incorporates rituals that facilitate icebreaking and ensure each participant interacts with different men each week. A study of a rural walking group found socialising before, during and after the walk was an essential highlight of the walk experience [31]. BMWT routinely facilitates conversation in pairs and small groups over coffee pre‐walk, during the walk, at the half‐way bridge stop and partner switch, and post‐walk in a covered seating area. Coffee is an icebreaker and is associated with expectations of conversation [43]. Having multiple conversations means walkers sooner meet people they like or have things in common with [44], accelerating feelings of comfort and enjoyment.

*Third place* has been associated with enhanced feelings of belonging and associated improvements to well‐being and reduced loneliness [3, 5, 47]. BMWT rituals repeatedly reinforce importance of the ‘group’, helping participants to feel they belong. Yellow‐Caps speak respectfully about the importance of caring for physical and mental well‐being. They remind participants of expectations of confidentiality (‘*what's said on the walk stays on the walk*’), thus communicating they are trusted group insiders. The ‘*Never walk alone*’ motto is repeated often, conspicuous on shirts and social media alongside a distinctive logo depicting a group of men, and also strictly adhered to on the walk. Each week walkers assemble as a group repeatedly, pre‐walk over coffee, during the briefing, at the bridge for a group photo and post‐walk—deepening belonging with regular attendance. A WhatsApp group and Facebook page keep the group connected between walks.

Rituals also mitigate the stress of uncertainty, allowing walks to run smoothly. The Yellow‐Cap leaders intervene rarely and discretely, but their presence reassures walkers that help is available if needed, which is perhaps especially important for a weekly event.

The study supports Oldenburg's [3] assertion that *third places* can feel more diverse from within than they appear from outside. BMWT walkers value diversity in fellow walkers' employment, education and social backgrounds, however the group are less ethnically and culturally diverse than the wider community. The diversity of *third places* highlighted by Dolley [21] needs to be critically examined to ensure they do not inadvertently replicate existing social hierarchies or exclude marginalised groups [27]. It is important also to reflect that older men and marginalised groups have diverse needs and preferences, and that BMWT is just one group‐based outlet or solution alongside others, such as sport or crafts.

The findings support claims that community‐based activities using a *third place* framework can nurture social interaction and feelings of belonging [18], and may play a positive role in rural men's mental health [9]. Many BMWT walkers have well‐being needs related to low social interaction and recent negative life events. Rural men often experience barriers to accessing mental health services, including cost, availability and stigma [1]. This study found that salutogenic *third place* features—welcoming, levelling, accessibility, conversation and playfulness—are potentially factors that support at‐risk men.

Based on the study findings, expansion of BMWT to increase availability is likely desirable. New times or venues should be planned carefully to preserve essential *Third Place* characteristics that contribute to its effectiveness, such as fostering conversation, and nurturing familiarity and belonging. Managing increased participant numbers requires planning to ensure the programme's health‐giving social interactions and supportive culture are maintained, particularly as leaders value the familiarity achievable in a smaller group. The Bathurst venue has considerable room for growth in numbers, but congestion and other path users should always be a consideration. Leaders should prioritise planning that includes crucial elements—such as pre‐walk coffee, walking in pairs and the half‐way stop—that facilitate connection and reinforce the group.

## 5 Limitations

The study sample was large as a proportion of BMWT walkers and leaders, but it was a sample of convenience reflecting views of active participants. There may have been a positivity bias resulting from a greater propensity among those who benefit from BMWT to be involved in the interviews. Similar to South et al.'s [48] walk group study, interviewees were very positive, and it may not capture perspectives from those who tried BMWT but did not return. The sample may be skewed toward those benefiting from BMWT, limiting insight into potential barriers. Two researchers walked regularly and became BMWT insiders, this may have produced a bias toward positivity in selection and interpretations [33], but most interviews with walkers were conducted by other interviewers, and findings were similarly positive for all five interviewers. It was noted in interviews and analysis that BMWT works well for those who continue to walk, but cannot cater for all. Future studies should explore perspectives of those who disengaged or haven't participated.

## 6 Conclusion

This study introduces *Third Place* as a framework for understanding and planning for rural men's participation in community well‐being programmes. It highlights an important overlap, that social and group factors influence feelings of enjoyment and belonging, and that social interaction and belonging are important aids to mental well‐being. BMWT is a group of men regularly walking together by a river, organised by volunteers and requiring relatively few resources. For this group of rural men, BMWT effectively uses culture and rituals to accentuate feelings of safety and inclusion, facilitate multiple conversations, and reinforce the group. For the rural men who participate, thoughtful rituals and a mind to characteristics of *third place* increase health‐giving social interaction and feelings of belonging. In rural areas where men in need often miss out on mental well‐being support, *Third Place* provides a framework to guide group and community planning.

## Author Contributions

**Peter Simmons:** funding acquisition, supervision, conceptualisation, methodology, investigation, data curation, and formal analysis, writing original draft preparation, review and editing. **Uchechukwu Levi Osuagwu:** supervision, conceptualisation, investigation, writing review and editing. **Hazel Dalton:** conceptualisation, investigation, writing review and editing. **Julaine Allan:** conceptualisation, writing review and editing. **John Eme:** investigation, data curation, formal analysis. **Tracy Macfarlane:** investigation, formal analysis. **Haider Mannan:** conceptualisation, data curation.

## Ethics Statement

Approval (H15627) was obtained from the Western Sydney University Human Research Ethics Committee, ensuring that all research procedures complied with Helsinki's Declaration for research on human participants.

## Conflicts of Interest

The authors declare no conflicts of interest.

## Supporting information


Appendix S1.

## Data Availability

The data that support the findings of this study are available from the corresponding author upon reasonable request.
