# Supplementary material for: Walk for Mental Well‐Being as a Third Place: A Qualitative Study of the Bathurst Men's Walk and Talk
Source: Aust J Rural Health. 2025 Jun 23;33(3):e70066. doi: 10.1111/ajr.70066 (PMC12183771; doi:10.1111/ajr.70066)
Supplement: Supplementary file 1 — Appendix S1. [file AJR-33-0-s001.docx]

**Appendix.**

**A** **BMWT interview guide (Prompt as required)**

1. **Orient and establish rapport - - Leaders and walkers**

Could you tell a little about yourself and your connection with Bathurst?

What is your association with BMW&T?

1. **Experiences and perceptions of BMWT**

……………………………………………………………………………………………………………………………

**Walker questions**

How did you come to be involved with BMW&T? (eg family, friend, advertising).

**Follow-up Qs**

What influenced you to participate in BMW&T

**Follow-up prompt**

(meet people, exercise, other?)

What do you like/not like about BMW&T?

**Follow-up Qs**

Is it a good idea that it is **men only**?

Do you **enjoy the conversations? (topics)**?

What about the **coffee** before the walk?

Do you enjoy **doing the same walk** each week, with **stop at half** way?

**Around 5kms walk**- is that OK?

Has BMW&T made a difference to your life at all? In what ways?

**Follow-up prompts**:

Have you made **new connections** or friends?

Has it **influenced the way you exercise**?

Has it led to **other activities and interactions**?

The walks often commence with a very **short talk about a health-related matter**. Do you think these are valuable?

**Follow-up prompt**

Have you **learnt about health or services available in the community**?

Do you think BMW&T has **influenced your mental wellbeing or thinking**? If so, how?

**Listening for: impacts - connections, activities, feelings (Physical, Mental, social)**

**Listening *for examples of important concepts eg ‘connection’, ‘belonging’, ‘confidence’, ‘mobility and fitness’, ‘stress’, ‘walking’, ‘exercise’.***

**……………………………………………………………………………………………………………………………….**

**Leader questions**

How did you come to be involved with BMW&T?

What do you feel is the purpose of BMW&T?

In terms of purpose, how is it going?

Have you seen any changes in walkers’ health or wellbeing?

What BMW&T features do you feel help to meet needs of the people who walk?

**Follow-up prompts**

What is the importance of structures and processes, the activities and rituals?

- Shirts
- Coffee van
- Chat before walk
- Stopping half-way

Is it important that it is men only?

What is importance of the venue – walk by river?

How are men recruited and why do you think they participate?

Do you perceive any barriers to participation in BMW&T?

**……………………………………………………………………………………………………………………………….**

1. **Future preferences for BMWT and Close**

What would you like to see for BMW&T for the future?
